# The direct and mediating effects of cognitive impairment on the occurrence of falls: a cohort study based on community-dwelling old adults

**DOI:** 10.3389/fmed.2023.1190831

**Published:** 2023-06-08

**Authors:** Tianyi Zhang, Cunmei Yang, Gangming Shu, Chang Gao, Hongying Ma, Lin Zou, Jing Zuo, Shaoni Liu, Jin Yan, Yixin Hu

**Affiliations:** ^1^Institution of Hospital Management, Department of Medical Innovation and Research, Chinese PLA General Hospital, Beijing, China; ^2^Geriatric Health Care Department 4th of The Second Medical Center and National Clinical Research Center for Geriatric Diseases, Chinese PLA General Hospital, Beijing, China; ^3^Geriatric Health Care Department 1st of The Second Medical Center, Chinese PLA General Hospital, Beijing, China; ^4^Graduate School of Chinese PLA General Hospital, Beijing, China

**Keywords:** cognitive impairment, falls, mediation effects, older people, epidemiology

## Abstract

**Background:**

Cognitive impairment has been reported to be associated with falls in older adults. However, the complex relationship among falls, cognitive impairment and its associated factors, which could be targeted with specific interventions, remains to be elucidated. This study aimed to examine the direct effects of cognitive impairment on falls, to identify the factors associated with cognitive impairment and to explore the mediation role of cognitive impairment in the association of fall with cognition related factors.

**Methods:**

This 1-year follow-up cohort study enrolled old adults aged 60  years or over. Information about demographic and anthropometric characteristics, fall outcomes, function and nutritional status were collected through face-to-face interview. Cognitive function was evaluated by the Montreal Cognitive Assessment (MoCA). Multivariable regression analyses were used to test the association between cognitive impairment and falls and to identify the factors related to cognitive impairment. Additionally, we conduct causal mediation analyses to estimate the mediation effects of cognitive impairment in the pathways of fall occurrence.

**Results:**

Of the 569 participants included in this study, 366 (64.32%) had cognitive impairment, 96 (16.87%) had fall history in the past 1  year, 81 (14.24%) experienced fall and 47 (8.26%) received treatment because of falling during the 1-year follow-up. The association between cognitive impairment and 1-year fall risk was confirmed after adjusting for multiple covariates [odds ratio (OR):2.03, 95% confidence interval (CI): 1.13–3.80]. IADL disability, depression and low grip strength were associated with a higher prevalence of cognitive impairment. While overweight, higher education and higher income level were found to be related to a lower risk of cognitive impairment. Among these associated factors, cognitive impairment mediated the positive association of falling with IADL ability and depression, and a negative relationship with education and income level.

**Conclusion:**

Our study not only confirmed the direct influence of cognitive impairment on fall risk in older adults, but also suggested a mediating role that cognitive impairment played in the pathways of fall occurrence. Our finding could help develop more specific interventions for fall prevention.

## Introduction

Falls have been an important serious public health problem since it can cause many adverse consequences, such as fracture, decreased quality of life, hospitalization and death, which have produced great health and economic burdens worldwide (1–3). The consequences of fall in older adults could be even worse for that it may not only lead to a reduction in functionality but also affect multiple aspects of life in older adults (4). It is estimated 30% of older people 65 or above could experience fall each year and this figure may continue to increase with the rising aging population globally (3, 5). Identifying the risk factors is efficient to prevent the occurrence of fall and further injuries through appropriate interventions.

To date, a number of factors have been identified to be associated with the risk of fall (6, 7). Among them, cognitive impairment is one of the crucial risk factors of falls among community-dwelling elderly adults (8–10). A large body of evidence has shown that there exists cognitive-motor interference phenomenon (11, 12). Cognitive function would impact walking and balance skills; while low physical function (11), such as sarcopenia and low muscle strength, is negatively associated with cognitive function (13, 14). The interdependent effects of cognitive and physical function have been of interest in rehabilitation field (12, 15). Furhtermore, fall is a complex event and is often companied with other geriatric syndromes and factors. Some geriatric syndromes, such as polypharmacy and multimorbidity, have been reported could affect both falls and cognitive function (16, 17). What is the relationship among cognitive impairment, falls, and other geriatric factors, how cognitive impairment increases the fall have not been fully elucidated yet. It has been reported that interventions directly on fall in patients with cognitive impairment, such as exercise to improve gait, balance and mobility, were insufficient to prevent fall occurrence (18). Interventions for cognitive impairment besides directly on fall would be more efficient in reducing fall incidence (19). However, management for cognitive impairment was limited in fall prevention (20). Identifying factors associated with cognitive impairment, knowing the relationship among these associated factors, cognitive impairment and fall, understanding the pathway through which cognitive impairment affect fall occurrence will provide more specific targets for interventions and thus increase the effectiveness of the intervention strategies.

Therefore, we carried out this cohort study in a Chinese retirement community to investigate the direct effect of cognitive impairment on falls, to identify the associated factors of cognitive impairment, and to explore the pathways by which cognitive impairment affects fall occurrence by using causal mediation analysis.

## Materials and methods

### Participants and study design

**Figure 1:**
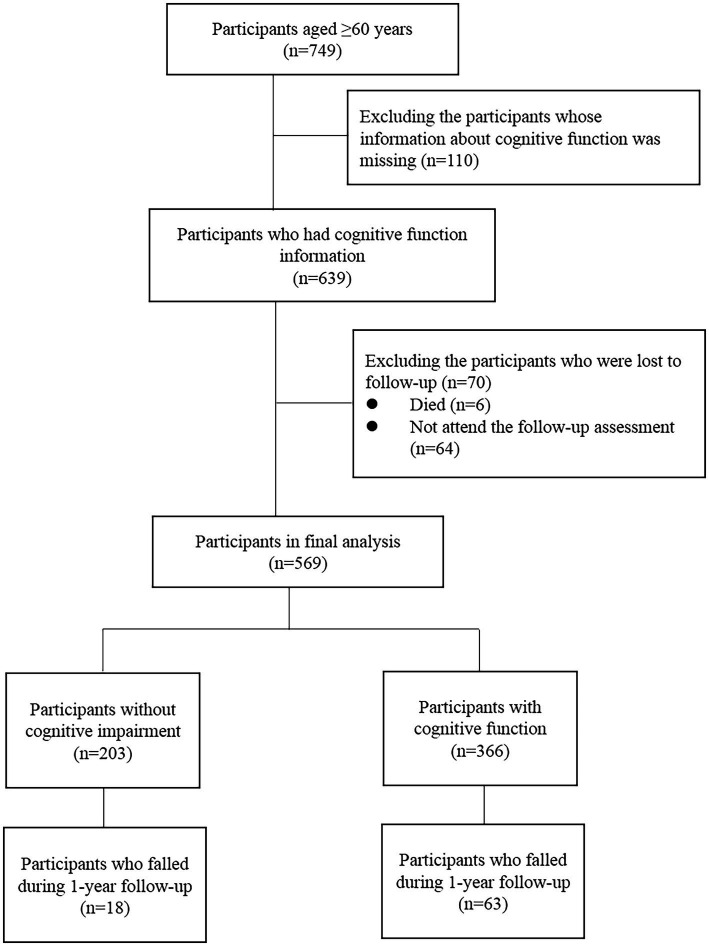
Flowchart of the study population.

The present study was a 1-year follow up cohort study. The inclusion criteria were: (1) age 60 years or over and (2) had electronic health records from long-run medical centers. The exclusion criteria were: (1) had a diagnosis of dementia by a dementia specialist; (2) a history of Parkinson’s disease; (3) terminal cancer. Based on these criteria, we recruited 749 old adults in a retirement community in Beijing from 2018 to 2019. After excluding the participants who did not receive physical or cognitive function assessment and who were lost to follow-up (including those who died and did not attend the follow-up assessment), 569 participants were included in the present analysis (Figure 1). This study has been approved by the Research Ethics Committee of Chinese PLA General Hospital (Ethic number: S2018–102-02) and all participants signed the informed consent.

### Cognitive function assessments

Cognitive function was assessed using the Montreal Cognitive Assessment (MoCA), which consists of eight domains including visuospatial skills and executive function (0–5 scores), functions (0–5 scores), delayed recall memory (0–5 scores), attention (0–6 scores), naming (0–3scores), language (0–3 scores), abstraction (0–2 scores),and orientation (0–6 scores). The total score of MoCA ranges from 0 to 30, with higher scores indicating higher cognitive function. Cognitive impairment was defined as MoCA scores of ≤25, ≤24 and ≤ 23 for participants aged 60–79, 80–89, and ≥ 90 years old, respectively (21).

### Falls

A fall was defined as an accidental event that caused a person unintentionally falls to the floor or a lower level, not because of intrinsic event (22). Falls information was obtained both at baseline and 1-year follow up. Participants or their spouses and children were asked if the participants had fallen down in the past 12 months. And if so at the 1-year follow up, they were also asked if the fall cause them to receive treatment.

### Covariates

Data regarding sociodemographic characteristics, lifestyle and anthropometric variables, chronic conditions and other health-related factors were collected in this study. Sociodemographic characteristics included sex, age (female, male), marital status (married, widowed or divorced), education (primary school or below, junior high school, senior high school, university or above) and household income monthly (≤5,000¥, 5,000¥–10,000¥, 10,000¥–15,000¥, >15,000¥). Lifestyle and anthropometric variables included smoking status (never, former and current smokers), being taken care of by others (no, yes), body mass index (BMI), calculated as body weight divided by height squared, classified as underweight (<18.5), normal (18.5–23.9), overweight (24.0–27.9), and obesity (≥28.0), waist to hip ratio and skeletal muscle mass index (SMI), calculated as appendicular skeletal muscle mass [measured using bioelectrical impedance analysis (BIA, InBody 270, Biospace Ltd., Seoul, Korea) divided by height squared]. Chronic conditions included activities of daily living (ADL) disabilities, instrumental activities of daily living (IADL) disabilities, chronic disease (including hypertension, coronary heart disease, diabetes, respiratory system disease, kidney disease), and polypharmacy. ADL and IADL disabilities were ascertained by the standardized questionnaires. Respondents who had difficulties in performing 1 or more ADL activities (bowel control, bladder control, grooming, toilet use, feeding, transfers, mobility, stairs, dressing, and bathing) were classified as having ADL disability (23), who had difficulties in social function related tasks (making/receiving phone calls, shopping, doing household chores, cooking meals, doing laundry, using transportation, taking medications and managing finances) were classified as IADL disability (24). Participants who had two or more diseases were defined as having comorbidities, who had taken five or more medicines were defined as having polypharmacy. The information on comorbidities and medications were acquired by participants, their spouses and children and through a careful review of medical documents. Health-related factors included status of depression, anxiety and nutrition, grip strength, gait speed and sleeping quality. Depression assessment was conducted using the 5-item geriatric depression scale (GDS-5) (25). Participants fulfilling two or more of the 5 conditions were defined as having a depressive tendency. Anxiety symptoms were assessed by 7-item general anxiety disorder scale (GAD-7) (26). Participants who got 5 or more points were defined as anxiety. Nutrition status was assessed using Mini Nutritional Assessment-short form (MNA-SF) (27). Participants who got a score of ≤11 were classified as having malnutrition or a risk of that. Grip strength was measured using a digital handgrip dynamometer (JAMAR Co., Ltd., United States). Low grip strength was defined as <26 kg in men and < 18 kg in women (28). Usual gait speed on a 6-meter course was measured objectively. Gait speed <0.8 m/s was classified as slow gait speed for both men and women (28). The quality of sleep was measured by Pittsburgh sleep quality index (PSQI) with a total point of 21 (29). Participants who got a score of >5 were defined as having poor quality.

### Statistical analysis

Baseline characteristics were described according to the status of cognitive function. Continuous variables were presented as median (the lower quartile, the upper quartile) and categorical variables as frequency and percentages (%). The difference in characteristics between the four groups was tested by student t test or rank sum test for continuous variables based on the normality of the data, and chi-square for categorical variables.

Logistic regression models were constructed to evaluate the association of fall with cognitive function [the odds ratios (ORs) and 95% confidence intervals (CIs) of cognitive impairment for the risk of fall compared with normal cognitive function was estimated. If 95% CI does not contain the value of 1, then it indicates a significant association between cognitive impairment and the risk of fall]. The initial models were crude without any adjustment (Model 1). The subsequent models included demographic and anthropometric variables, such as sex, age, education, income, BMI, waist to hip ratio, SMI (Appendicular Skeletal Muscle Mass Index) as adjustments (Model 2). In the Model 3, function and nutritional status were added, such as the status of depression, anxiety, nutrition, ADL and IADL limitations. Further, considering the bias that may be brought by the limited sample size and the imbalance of the outcome, we introduced firth logistic regression (30) to estimate the association (Model 4), adjusting the covariates in the Model 3.

To further explore the role in the pathways of fall occurrence, we firstly constructed univariate and multivariate-adjusted binary logistic models to identify the factors associated with cognitive function. Then, we conducted causal mediation analyses with the counterfactual framework proposed by Imai et al. (31) were conducted to determine whether the cognitive status can explain or mediated the relationship between the identified associated factors and the risk of fall, after adjusting for the covariates listed in model 3. Pure natural indirect effect (PNIE), the expected difference between two counterfactual outcomes for the same individual whose mediator differs from the value that would had been observed under exposure value x^*^ to that under exposure value x while holding exposure at the level of x, E(Y_i_(x,M(x^*^)) − Y_i_(x,M(x))), total natural indirect effect (TNIE), the expected difference between two counterfactual outcomes for the same individual whose mediator differs from the value that would had been observed under exposure value x^*^ to that under exposure value x while holding exposure at the level of x^*^, E(Y_i_(x^*^,M(x^*^)) − Y_i_(x^*^,M(x))), pure natural direct effect (PNDE), the expected difference between two counterfactual outcomes for the same individual whose exposure value differed from x^*^ to x, while holding mediator constant at its potential value under exposure value of x, E(Y_i_(x^*^,M(x)) − Y_i_(x,M(x))), total natural direct effect (TNDE), the expected difference between two counterfactual outcomes for the same individual whose exposure value differed from x^*^ to x, while holding mediator constant at its potential value under exposure value of x^*^, E(Y_i_(x^*^,M(x^*^)) − Y_i_(x,M(x^*^))), average causal mediation effect (ACME, the average of PNIE and TNIE), average direct effect (ADE, the average of PNDE and TNDE) (32) and their 95% CIs were estimated in the causal mediation analyses. If 95% CI does not contain the value of 0, then it indicates the effect is significant.

For missing data, we firstly conducted complete data analysis after excluding all missing observations. Further, we performed multiple imputations for missing values and created five imputed datasets. We combined the results of the analyses of imputations as sensitivity analysis.

All reported *p* values were two-sided, with statistical significance deemed at *p* < 0.05. Data analyses were conducted using R software, version 4.0.2.

## Results

Among the 569 participants included in this study, 366 (64.32%) had cognitive impairment, 96 (16.87%) and 81 (14.24%) experienced fall in the past 1 year and during the 1-year follow-up respectively, and 47 (8.26%) needed and received treatment because of fall during the 1-year follow-up. Table 1 summarized and compared characteristics of the participants between participants who fell and those who did not during 1-year follow-up. Participants who fell during the 1-year follow-up were more likely to have poor sleep quality (50.62% vs. 34.63%, *p* = 0.0034), depression (19.75% vs. 9.84%, *p* = 0.0285) and cognitive impairment (77.78% vs. 62.09%, *p* = 0.0064).

**Table 1:** Baseline characteristics between 1-year follow-up fallers and none-fallers group.

	Fall		*p*-value
	No (*N* = 488)	Yes (*N* = 81)	
Age (years)	86 (82–88)	86 (82–89)	0.9498
Male (%)	191 (39.14)	29 (35.80)	0.5683
Education
Primary school or below	36 (7.38)	6 (7.41)	0.6620
Junior high school	80 (16.39)	20 (24.69)	
Senior high school	135 (27.66)	13 (16.05)	
University or above	237 (48.57)	42 (51.85)	
Widowed or divorced (%)	182 (37.30)	30 (37.04)	0.9645
Being taken care of by others (%)	372 (76.23)	58 (71.60)	0.3701
Household income monthly
≤5,000¥	85 (17.42)	12 (14.81)	0.6098
5,000¥–10,000¥	127 (26.02)	20 (24.69)	
10,000¥–15,000¥	62 (12.70)	13 (16.05)	
>15,000¥	214 (43.85)	36 (44.44)	
Smoking status
Never	405 (82.99)	65 (80.25)	0.8326
Former	73 (14.96)	14 (17.28)	
Current	10 (2.05)	2 (2.47)	
BMI (kg/m^2^)	24.05 (21.98–26.21)	23.59 (21.15–26.33)	0.6350
Underweight	213 (43.65)	39 (48.15)	0.7992
Normal	24 (4.92)	4 (4.94)	
Overweight	197 (40.37)	28 (34.57)	
Obesity	54 (11.07)	10 (12.35)	
Waist to hip ratio	0.89 (0.84–0.94)	0.88 (0.83–0.94)	0.7401
SMI	6.40 (5.80–7.10)	6.50 (5.80–7.30)	0.9641
Grip strength (kg)	23.00 (19.10–28.20)	22.65 (18.90–26.70)	0.4211
Decrease in grip strength	192 (39.34)	30 (37.04)	0.5396
Gait speed (m/s)	0.88 (0.73–1.05)	0.87 (0.67–1.00)	0.1266
Decrease in gait speed	111 (22.75)	21 (25.93)	0.1737
ADL disability (%)	173 (35.45)	40 (49.38)	0.0552
IADL disability (%)	265 (54.30)	44 (54.32)	0.3485
Anxiety (%)	49 (10.04)	11 (13.58)	0.5983
Depression (%)	48 (9.84)	16 (19.75)	0.0285
Poor sleep quality (%)	169 (34.63)	41 (50.62)	0.0034
Malnutrition (%)	59 (12.09)	11 (13.58)	0.6241
Chronic disease
Hypertension (%)	346 (71.34)	55 (70.51)	0.8810
Coronary heart disease (%)	238 (48.77)	48 (59.26)	0.2042
Diabetes (%)	139 (28.48)	26 (32.10)	0.7437
Respiratory system disease (%)	93 (19.06)	17 (20.99)	0.7524
Digestive system disease (%)	219 (44.88)	37 (45.68)	0.9209
Kidney disease (%)	57 (11.68)	7 (8.64)	0.4718
Cognitive impairment	303 (62.09)	63 (77.78)	0.0064
Comorbidity (%)	416 (85.25)	69 (85.19)	0.3043
Polypharmacy (%)	143 (29.30)	21 (25.93)	0.5346

### Factors associated with cognitive impairment

The multivariate logistic regression showed the following factors were associated with increased prevalence of cognitive impairment: being cared by others (OR: 2.52, 95%CI: 1.43–4.45), IADL disability (OR: 2.22, 95%CI: 1.28–3.85), depression (OR: 2.85, 95%CI: 1.16–6.99) and low grip strength (OR: 2.33, 95%CI: 1.29–4.22; Table 2). While, some factors were found to be negatively associated with cognitive impairment prevalence, including higher household income (10000–15,000 vs. ≤5,000 ¥ per month, OR: 0.29, 95%CI: 0.11–0.79, >15,000 vs. ≤5,000 ¥ per month: OR: 0.30, 95%CI: 0.10–0.86) and overweight (OR: 0.52, 95%CI: 0.28–0.98; Table 2). These associations were confirmed in the sensitivity analysis, except the associations of cognitive impairment with household income. Additionally, higher education was found to be associated with the lower prevalence of cognitive impairment in the sensitivity analysis (Senior high school vs. Primary school or below: OR: 0.22, 95%CI: 0.07–0.71; University or above vs. Primary school or below: OR: 0.27, k95%CI: 0.09–0.84; Table 3).

**Table 2:** Association of cognitive impairment with other factors in the old adults of this study.

	Univariate analysis		Multivariate analysis	
	OR (95% CI)	*p*	OR (95% CI)	*p*
Age(years)	1.05 (1.02, 1.08)	0.0009	1.02 (0.96, 1.07)	0.5579
Sex, female versus male	1.23 (0.87, 1.75)	0.2422	1.78 (0.71, 4.47)	0.2168
Education
Junior high school versus primary school or below	0.48 (0.17, 1.37)	0.1687	0.47 (0.12, 1.82)	0.2734
Senior high school versus primary school or below	0.19 (0.07, 0.50)	0.0009	0.28 (0.07, 1.04)	0.0576
University or above versus primary school or below	0.20 (0.07, 0.51)	0.0009	0.34 (0.09, 1.25)	0.1040
Widowed or divorced versus married	0.68 (0.47, 0.97)	0.0357	1.03 (0.60, 1.77)	0.9104
Taken care of by others	2.27 (1.53, 3.35)	<0.0001	2.52 (1.43, 4.45)	0.0015
Household income monthly
5,000¥–10,000¥ versus ≤5,000¥	1.08 (0.62, 1.89)	0.7797	0.76 (0.32, 1.84)	0.5468
10,000¥–15,000¥ versus ≤5,000¥	0.46 (0.25, 0.86)	0.0148	0.29 (0.11, 0.79)	0.0152
>15,000¥ versus ≤5,000¥	0.76 (0.46, 1.25)	0.2738	0.30 (0.10, 0.86)	0.0258
Smoking status
Former versus never	1.08 (0.67, 1.74)	0.7632	1.65 (0.76, 3.59)	0.2031
Current versus never	1.70 (0.45, 6.36)	0.4312	8.07 (0.81, 80.46)	0.0751
BMI(kg/m2)
Underweight versus normal	2.43 (0.89, 6.60)	0.0829	0.80 (0.11, 5.99)	0.8306
Overweight versus normal	0.76 (0.53, 1.11)	0.1522	0.52 (0.28, 0.98)	0.0416
Obesity versus normal	1.25 (0.69, 2.27)	0.4648	0.69 (0.24, 1.94)	0.4796
Waist to hip ratio	0.85 (0.10, 6.97)	0.8831	3.67 (0.09, 143.31)	0.4865
SMI	0.95 (0.84, 1.07)	0.4169	1.13 (0.76, 1.68)	0.5504
ADL disability	1.77 (1.22, 2.56)	0.0024	1.37 (0.82, 2.31)	0.2307
IADL disability	3.40 (2.37, 4.88)	<0.0001	2.22 (1.28, 3.85)	0.0044
Anxiety	1.48 (0.82, 2.67)	0.1958	0.85 (0.33, 2.19)	0.7291
Depression	2.77 (1.44, 5.33)	0.0023	2.85 (1.16, 6.99)	0.0218
Poor sleep quality	1.42 (0.98, 2.04)	0.0619	1.20 (0.72, 1.99)	0.4873
Malnutrition	2.22 (1.22, 4.04)	0.0092	3.96 (0.95, 16.51)	0.0591
Comorbidity	1.02 (0.58, 1.80)	0.9356	0.85 (0.37, 1.96)	0.7010
Polypharmacy	1.33 (0.90, 1.96)	0.1474	1.13 (0.64, 2.00)	0.6768
Fall history in the last year	1.16 (0.73, 1.86)	0.5257	0.75 (0.38, 1.48)	0.4135
Low gait speed	2.50 (1.58, 3.95)	<0.0001	1.55 (0.83, 2.89)	0.1725
Low grip strength	2.56 (1.75, 3.73)	<0.0001	2.33 (1.29, 4.22)	0.0050

**Table 3:** Association of cognitive impairment with other factors in the old adults in the sensitivity analysis.

	Univariate analysis		Multivariate analysis	
	OR (95% CI)	*p*	OR (95% CI)	*p*
Age(years)	1.05 (1.02, 1.08)	0.0009	1.03 (0.99, 1.08)	0.1552
Sex, female versus male	1.23 (0.87, 1.75)	0.2422	1.92 (0.88, 4.20)	0.1031
Education
Junior high school versus primary school or below	0.48 (0.17, 1.37)	0.1687	0.59 (0.18, 1.90)	0.3781
Senior high school versus primary school or below	0.19 (0.07, 0.50)	0.0009	0.22 (0.07, 0.71)	0.0106
University or above versus primary school or below	0.20 (0.07, 0.51)	0.0009	0.27 (0.09, 0.84)	0.0240
Widowed or divorced versus married	0.68 (0.47, 0.97)	0.0357	0.91 (0.58, 1.43)	0.6732
Taken care of by others	2.27 (1.53, 3.35)	<0.0001	1.98 (1.22, 3.21)	0.0058
Household income monthly
5,000¥–10,000¥ versus ≤5,000¥	1.08 (0.62, 1.89)	0.7797	1.28 (0.63, 2.60)	0.4979
10,000¥–15,000¥ versus ≤5,000¥	0.46 (0.25, 0.86)	0.0148	0.45 (0.20, 1.03)	0.0584
>15,000¥ versus ≤5,000¥	0.76 (0.46, 1.25)	0.2738	0.51 (0.22, 1.20)	0.1256
Smoking status
Former versus never	1.08 (0.67, 1.74)	0.7632	1.53 (0.82, 2.84)	0.1809
Current versus never	1.70 (0.45, 6.36)	0.4312	2.38 (0.55, 10.26)	0.2435
BMI(kg/m^2^)
Underweight versus normal	2.43 (0.89, 6.60)	0.0829	3.15 (0.76, 12.99)	0.1132
Overweight versus normal	0.76 (0.53, 1.11)	0.1522	0.55 (0.31, 0.96)	0.0345
Obesity versus normal	1.25 (0.69, 2.27)	0.4648	0.69 (0.28, 1.71)	0.4274
Waist to hip ratio	0.98 (0.11, 8.54)	0.9880	8.67 (0.26, 292.73)	0.2284
SMI	0.95 (0.85, 1.07)	0.4307	1.25 (0.88, 1.79)	0.2153
ADL disability	1.77 (1.22, 2.55)	0.0024	1.21 (0.78, 1.88)	0.3885
IADL disability	3.44 (2.40, 4.93)	<0.0001	2.51 (1.57, 4.03)	0.0001
Anxiety	1.39 (0.77, 2.48)	0.2722	0.70 (0.34, 1.44)	0.3305
Depression	2.57 (1.38, 4.80)	0.0031	2.61 (1.23, 5.53)	0.0125
Poor sleep quality	1.40 (0.97, 2.02)	0.0689	1.30 (0.84, 2.01)	0.2398
Malnutrition	2.24 (1.23, 4.09)	0.0081	1.17 (0.48, 2.81)	0.7312
Comorbidity	0.99 (0.56, 1.75)	0.979	0.72 (0.35, 1.46)	0.3599
Polypharmacy	1.33 (0.90, 1.96)	0.1474	1.13 (0.70, 1.84)	0.6167
Fall history in the last year	1.16 (0.73, 1.84)	0.5336	0.82 (0.47, 1.42)	0.4758
Low gait speed	2.63 (1.67, 4.15)	<0.0001	1.48 (0.86, 2.52)	0.1550
Low grip strength	2.61 (1.79, 3.81)	<0.0001	2.12 (1.29, 3.49)	0.0030

### Relationships between cognitive impairment and fall

Compared to those without cognitive impairment, participants with cognitive impairment had a higher incidence of fall during the 1-year follow-up (17.21% vs. 8.87%). After adjusting for multiple confounders and considering the imbalance of the outcome, cognitive impairment was associated with the increased risk of fall during the 1-year follow-up (OR: 2.03, 95%CI: 1.13–3.80; Table 4, Model 4). However, the association of cognitive impairment was non-significant with the fall in the past year (OR: 0.83, 95%CI: 0.49–1.41; Table 4, Model 4) and the treatment needed and received because of fall during the 1-year follow-up (OR: 1.25, 95%CI: 0.58–2.70; Table 4, Model 4).Similar results could also be observed in the sensitivity analysis based on multiple imputed data (Table 5).

**Table 4:** Associations between cognitive impairment and fall.

	Model 1 OR (95% CI)	Model 2 OR (95% CI)	Model 3 OR (95% CI)	Model 4 OR (95% CI)
Fall in the last year	1.16 (0.73, 1.86)	1.05 (0.63, 1.74)	0.77 (0.44, 1.33)	0.83 (0.49, 1.41)
Fall during the 1-year follow-up	2.14 (1.23, 3.72)	2.31 (1.28, 4.19)	2.25 (1.19, 4.23)	2.03 (1.13, 3.80)
Treatment required because of fall during the 1-year follow-up	1.74 (0.88, 3.43)	1.77 (0.85, 3.71)	1.33 (0.53, 3.32)	1.25 (0.58, 2.70)

Model 1 was crude without adjustments. Model 2 was multivariate logistic regression adjusted for age, sex, education, household incomes, marital status, whether being taken care of by others, smoking status, BMI, waist to hip ratio and SMI. Model 3 was Model 2 plus ADL and IADL disability, anxiety, depression, sleep quality, nutrition status, grip strength, gait speed, comorbidity and polypharmacy. Model 4 was firth logistic regression adjusted for covariates in the model 3.

**Table 5:** Associations between cognitive impairment and fall in the sensitivity analysis.

	Model 1 OR (95% CI)	Model 2 OR (95% CI)	Model 3 OR (95% CI)	Model 4 OR (95% CI)
Fall in the last year	1.16 (0.73, 1.86)	1.05 (0.63, 1.74)	0.77 (0.44, 1.33)	0.83 (0.49, 1.41)
Fall during the 1-year follow-up	2.14 (1.23, 3.72)	2.31 (1.28, 4.19)	2.25 (1.19, 4.23)	2.03 (1.13, 3.80)
Treatment required because of fall during the 1-year follow-up	1.74 (0.88, 3.43)	1.82 (0.88, 3.78)	1.75 (0.81, 3.79)	1.73 (0.82, 3.55)

Model 1 was crude without adjustments. Model 2 was multivariate logistic regression adjusted for age, sex, education, household incomes, marital status, whether being taken care of by others, smoking status, BMI, waist to hip ratio and SMI. Model 3 was Model 2 plus ADL and IADL disability, anxiety, depression, sleep quality, nutrition status, grip strength, gait speed, comorbidity and polypharmacy. Model 4 was firth logistic regression adjusted for covariates in the model 3.

### Causal mediation analysis

**Figure 2:**
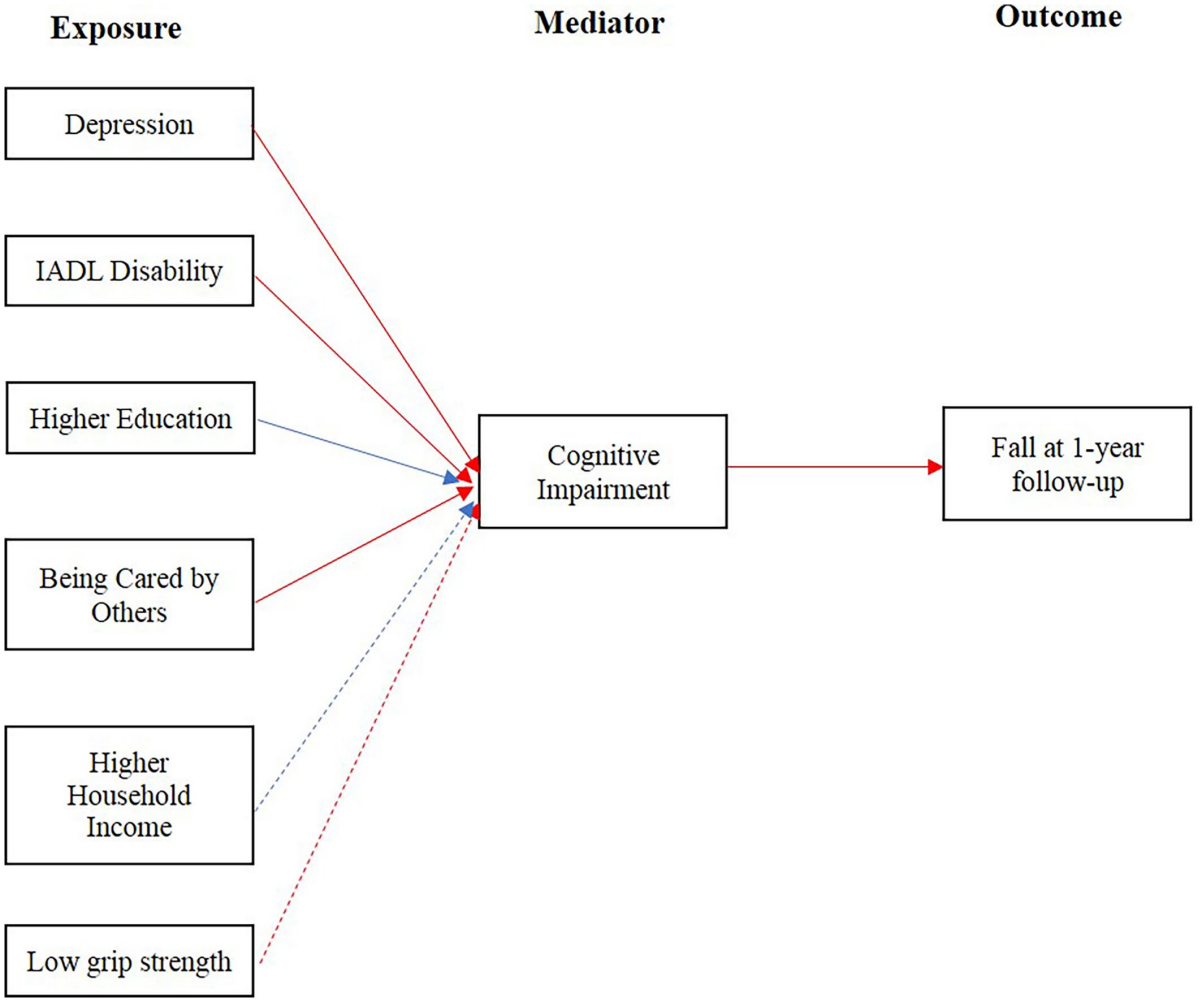
Causal diagram of relation among cognitive impairment associated factors, cognitive impairment and fall at 1-year follow-up. Red arrow lines represented the mediator mediated a positive association between exposure and outcome. Blue arrow lines represented the mediator mediated a negative association between exposure and outcome. Solid arrow lines represented the mediation associations were also shown in the sensitivity analyses. Dashed arrow lines represented the mediation associations were not confirmed in the sensitivity analyses.

Besides the direct effect of cognitive impairment on fall during 1-year follow-up, it also mediated the positive association between the risk of fall and depression (ACME: 2.05, 95%CI: 0.10–4.62%), IADL disability (ACME: 1.20, 95%CI: 0.13–3.74%), being taken care of by others (ACME: 1.28, 95%CI: 0.10–4.04%; Table 6; Figure 2; Supplementary Figure S1). Further, higher household income and education may decrease the risk of fall with cognitive function as the mediator (Table 6; Figure 2; Supplementary Figure S1). All these mediation effects were also shown in the sensitivity analysis except for higher household income (Table 7; Figure 2; Supplementary Figure S2). In addition, cognitive impairment was also found to mediate the association of the increased risk of fall with low grip strength (Table 7; Figure 2; Supplementary Figure S2).

**Table 6:** Mediating effects of cognitive impairment in the association of fall during 1 year follow up and the other factors in the old adults.

Exposure	Indirect Effect			Direct Effect		
	PNIE (95% CI)	TNIE (95% CI)	ACME (95% CI)	PNDE (95% CI)	TNDE (95% CI)	ADE (95% CI)
Depression	1.76% (0.05, 3.96%)	2.33% (0.12, 5.22%)	2.05% (0.10, 4.62%)	5.69% (−4.24, 19.54%)	6.26% (−4.38, 20.80%)	5.98% (−4.44, 20.05%)
IADL Disability	1.32% (0.14, 4.30%)	1.08% (0.11, 3.18%)	1.20% (0.13, 3.74%)	−3.44% (−11.53, 4.41%)	−3.68% (−12.70, 4.49%)	−3.56% (−12.12, 4.47%)
Low girp strength	1.13% (−0.01, 3.13%)	1.12% (−0.02, 3.01%)	1.13% (−0.03, 3.01%)	−0.01% (−7.21, 8.55%)	−0.01% (−7.85, 8.73%)	−0.01% (−7.40, 8.60%)
Senior high school versus Primary school or below	−1.64% (−6.23, −0.05%)	−1.37% (−4.33, −0.10%)	−1.50% (−5.08, −0.14%)	−3.06% (−21.12, 9.40%)	−2.79% (−20.12, 8.32%)	−2.92% (−20.73, 8.81%)
University or above versus Primary school or below	−1.55% (−5.47, −0.03%)	−1.58% (−4.74, −0.08%)	−1.56% (−4.80, −0.08%)	0.39% (−16.92, 12.71%)	0.36% (−15.66, 11.72%)	0.38% (−16.42, 12.15%)
Household income between 10,000¥ and 15,000¥ monthly versus ≤5,000¥ monthly	−1.01% (−4.16, −0.11%)	−1.76% (−6.41, −0.19%)	−1.39% (−4.64, −0.11%)	8.97% (−4.56, 22.64%)	8.22% (−4.84, 20.10%)	8.59% (−4.43, 21.29%)
Household income >15,000¥ monthly versus ≤5,000¥ monthly	−1.05% (−4.20, −0.08%)	−1.62% (−5.67, −0.02%)	−1.33% (−4.43, −0.02%)	6.87% (−8.84, 16.30%)	6.30% (−7.91, 14.63%)	6.59% (−8.54, 15.15%)
BMI overweight versus BMI normal	−0.69% (−2.76, 0.07%)	−0.63% (−2.53, 0.05%)	−0.66% (−2.57, 0.08%)	−1.95% (−11.50, 5.22%)	−1.88% (−11.23, 4.86%)	−1.91% (−11.30, 5.04%)
Being taken care of by others	1.53% (0.20, 5.36%)	1.04% (0.10, 3.29%)	1.28% (0.10, 4.04%)	−7.39% (−16.58, 1.19%)	−7.88% (−17.55, 1.22%)	−7.63% (−17.02, 1.20%)

The causal mediation analysis was adjusted by sex, age, smoking status, BMI, waist to hip ratio, SMI, ADL and IADL disability, anxiety, depression, sleep quality, nutrition status, grip strength, gait speed, comorbidity and polypharmacy.

**Table 7:** Mediating effects of cognitive impairment in the association of fall during 1 year follow up and the other factors in the old adults in the sensitivity analysis.

Exposure	Indirect Effect			Direct Effect		
	PNIE (95% CI)	TNIE (95% CI)	ACME (95% CI)	PNDE (95% CI)	TNDE (95% CI)	ADE (95% CI)
Depression	1.26% (0.09, 2.93%)	1.77% (0.16, 4.13%)	1.52% (0.13, 3.48%)	10.16% (0.13, 22.07%)	10.67% (0.14, 23.02%)	10.42% (0.13, 22.54%)
IADL disability	1.46% (0.15, 3.43%)	1.20% (0.12, 2.66%)	1.33% (0.14, 3.03%)	−4.01% (−11.24, 3.46%)	−4.28% (−11.98, 3.69%)	−4.14% (−11.63, 3.57%)
Low girp strength	1.09% (0.08, 2.61%)	0.90% (0.07, 2.11%)	1.00% (0.07, 2.30%)	−3.19% (−9.79, 3.92%)	−3.38% (−10.43, 4.25%)	−3.28% (−10.01, 3.97%)
Senior high school versus Primary school or below	−2.17% (−4.94, −0.23%)	−1.53% (−3.43, −0.15%)	−1.85% (−3.96, −0.20%)	−7.74% (−23.68, 6.16%)	−7.10% (−22.30, 5.48%)	−7.42% (−23.01, 5.84%)
University or above versus Primary school or below	−1.88% (−4.42, −0.18%)	−1.75% (−4.02, −0.18%)	−1.81% (−4.20,-0.18%)	−2.61% (−19.46, 9.53%)	−2.48% (−18.34, 8.72%)	−2.54% (−18.91, 9.11%)
Household income between 10,000¥ and 15,000¥ monthly versus ≤5,000¥ monthly	−0.86% (−2.33, 0.06%)	−1.32% (−3.48, 0.09%)	−1.09% (−2.79, 0.07%)	8.47% (−2.53, 20.15%)	8.01% (−2.36, 19.21%)	8.24% (−2.43, 19.65%)
Household income >15,000¥ monthly versus ≤5,000¥ monthly	−0.68% (−2.16, 0.31%)	−1.05% (−3.25, 0.49%)	−0.87% (−2.64,0.41%)	8.26% (−2.00, 17.23%)	7.89% (−1.94, 16.38%)	8.07% (−1.96, 16.84%)
BMI overweight versus BMI normal	−0.69% (−1.89, 0.17%)	−0.65% (−1.90, 0.16%)	−0.67% (−1.87,0.16%)	−1.62% (−8.30, 5.47%)	−1.58% (−8.05, 5.16%)	−1.60% (−8.14, 5.32%)
Being taken care of by others	1.19% (0.03, 2.92%)	0.90% (0.00, 2.10%)	1.04% (0.02, 2.50%)	−6.68% (−15.13, 0.66%)	−7.88% (−17.55, 1.22%)	−6.83% (−15.43, 0.68%)

The causal mediation analysis was adjusted by sex, age, smoking status, BMI, waist to hip ratio, SMI, ADL and IADL disability, anxiety, depression, sleep quality, nutrition status, grip strength, gait speed, comorbidity and polypharmacy.

## Discussion/conclusion

To our best knowledge, this was the first study to explore the mediation effect of cognitive impairment on the occurrence of fall. Based on the 1-year follow-up cohort of older adults living in a Chinese retirement community with regular health assessments and education, we confirmed the positive association between cognitive impairment and subsequent fall. Furthermore, despite the direct effect on fall occurrence, cognitive impairment also mediated the association of fall with IADL disability, depression, education, household income and grip strength (Figure 2).

The incidence of fall during the 1-year follow-up was 14.24%, lower than that reported in previous studies (33–35), which may be owing to the high prevalence of being cared by others (75.57%). However, the fall rate was still in a relatively high level, which highlights the importance of preventing fall occurrence and significance of our study.

Compatible with previous findings (8, 35), we found a strong association between cognitive impairment and fall during 1-year follow up after adjusting multiple covariates. However, the association of cognitive impairment with fall based on cross-sectional design and with treatment due to fall at 1-year were not observed in this study. Several studies have reported a positive association between cognitive impairment and fall in a cross-sectional analysis (36, 37). While, study by Ma et al. showed cognitive impairment only did not associated higher prevalence of falls based on cross-sectional design (38). These inconsistencies may come from the difference in tools of assessing cognitive function and the nature of cross-sectional design which measures the outcome and exposure at the same time and cannot determine the cause and effect. The association between cognitive impairment and severe fall, such as fall related injuries, was also controversial. Lee et al. reported that cognitive impairment was associated with fall-related injury among older adults (39). Tsutsumimoto et al. observed a positive association of cognitive impairment existed with fall, but not with fall-related fracture (9). This discrepancy might be resulted from that the fall outcomes were self-reported and the definition of fall related injury may be different among responders. Further, the reason that we did not find the association between cognitive impairment and fall that need treatment could also be attributed to the high prevalence of receiving care (81.15%) in participants with cognitive impairment, which might mitigated the severity of the consequences. Future studies are needed to verify this speculation.

In our study, we also found IADL disability, depression and low grip strength were associated with higher risk of cognitive impairment. Participants who were overweight and had higher education and income level were less likely to suffer from cognitive impairment. These findings were consistent with previous studies (40–46). Also we found there was a positive relationship between cognitive impairment and being cared by others, which may be because that participants with cognitive impairment were more likely to have IADL disability and thus need care from others. Similar phenomena were also reported by Maria et al. who found cognitive impairment elderly outpatients were associated with a high prevalence of being accompanied at medical consultation (47).

Despite the direct effects of cognitive impairment on fall, we also found the indirect and mediation effect of cognitive impairment on the occurrence of fall. Cognitive impairment mediated the association between IADL disability, low grip strength and fall, which means IADL disability and low grip strength could increase the risk of fall indirectly by increasing the risk of cognitive impairment. These findings can partially explained that the intervention strategies only focused on physical function cannot prevent fall effectively and management for cognitive impairment is essential for fall prevention. We also found cognitive impairment mediated the association between depression and fall. The relationship between depression, cognitive impairment and the risk of fall was complex and remained unclear. Roh et al. suggested depression and cognitive impairment had a synergistic effect on the risk of fall (48). The mediation effect of cognitive impairment may contributed to this synergistic effect, as depression can worsen the cognitive function and further increase the fall risk. In addition, higher education and household income were found to decrease the risk of fall indirectly through the mediation effect of cognitive impairment. Education and income, as components of socioeconomic status (SES), are considered as one of the main causes of health disparities among different population groups, but it does not affect the health outcome directly (49). Thus, mediated by cognitive impairment could be one of pathways that SES exerts its effect on the fall occurrence. Furthermore, although an indirect association of being cared by others with fall was observed in our study, this may be not resulted from the effect of care from others, but due to the strong association of being cared with cognitive impairment. Cares itself had a tendency to decrease the risk of fall directly, though this effect was not significant. Further investigations were indispensable to study the effect of cares on the fall.

The strengths of this study includes the use of MoCA scale to assess cognitive impairment, which had good rest-retest reliability and was more comprehensive and sensitive than other cognition measures (50). Furthermore, this is the first study to investigate the association among fall, cognitive impairment and its associated factors based on community-dwelling old adults cohort using causal mediation analysis, which provides evidence to clarify the role of cognitive impairment in the pathways of fall occurrence. Causal mediation analysis has been gaining popularity during the last decade, which is preferred on traditional mediation analysis as it provides causal effect definitions (32, 51–53). However, most studies that used causal mediation analysis reported the proportion mediated as effect size measure. Although using proportion mediated as effect size measure has an intuitive interpretation, it suffers important limitations especially when the sample size is limited and the directions of direct and indirect effect size are opposite (32). The natural indirect effect estimate with a confidence interval is more recommended (32). So in our study, we substituted PNIE, TNIE and ACME, for proportion mediated.

There are also some limitations in our study. First, the factors associated with cognitive impairment were identified based on cross-sectional design which was less informative for causal inference. Future studies with longer follow-up and measurements of these factors at each wave will be required to elucidate the relationship between cognitive impairment and its associated factors, and the mediating role of cognitive impairment in the association of these factors with fall. Second, the participants were enrolled from a retirement with a convenience sampling process and the income and education level of the participants were relatively high, which might underestimate the observed association and limit the generalizability of our study results. Finally, the sample size of this study was relatively small. However, we used firth logistic regression to handle the bias that might be brought by limited sample size.

In conclusion, our study confirmed the positive association between cognitive impairment and fall risk based on a cohort from community-dwelling old adults with regular health assessments and education. Additionally, we suggested that cognitive impairment played a mediator in the pathways of fall occurrence, beside its direct influence on fall risk. Our study could provide basis for clarifying the role of cognitive impairment in the fall occurrence and would be helpful to designing more specific interventions for fall prevention.

## Data availability statement

The raw data supporting the conclusions of this article will be made available by the authors, without undue reservation.

## Ethics statement

The studies involving human participants were reviewed and approved by Research Ethics Committee of Chinese PLA General Hospital (Ethic number: S2018-102-02). The patients/participants provided their written informed consent to participate in this study.

## Author contributions

TZ performed statistical analyses, wrote the first draft of the manuscript. YH involved in the study design, develop the investment in community, prepare the data, interpreting the results, revise the first draft of the manuscript. CY, GS, CG, HM, LZ, JZ, SL, and JY performed the investment in community and prepare the data. All authors read and approved the final manuscript, thereby taking full responsibility for the work and manuscript content.

## Funding

YH are sponsored by Healthcare Fund (20BJZ30), Foundation of National Clinical Research Center for Geriatric Diseases (NCRCG-PLAGH-2022008).

These funding bodies provided the support of the design of the study and collection, analysis, and interpretation of data and the writing of the manuscript.

## Conflict of interest

The authors declare that the research was conducted in the absence of any commercial or financial relationships that could be construed as a potential conflict of interest.

## Publisher’s note

All claims expressed in this article are solely those of the authors and do not necessarily represent those of their affiliated organizations, or those of the publisher, the editors and the reviewers. Any product that may be evaluated in this article, or claim that may be made by its manufacturer, is not guaranteed or endorsed by the publisher.

## Supplementary material

SUPPLEMENTARY FIGURE S1 The causal mediation analyses for fall, cognitive impairment and its associated factors.

SUPPLEMENTARY FIGURE S2 The causal mediation analyses for fall, cognitive impairment and its associated factors after multiple imputation.

## References

[1] World Health Organization. Evidence Profile: Risk of Falls - Integrated Care for Older People (2017). Geneva, Switzerland.

[2] C. PHAo. Public Health Agency of Canada. Seniors’ Falls in Canada: Second Report, (2014). Ottawa.

[3] World Health Organization. World Health Organization. World report on ageing and health, (2015). Geneva, Switzerland.

[4] Giovannini S Brau F Galluzzo V Santagada D Loreti C Biscotti L. Falls among older adults: screening, identification, rehabilitation, and management. Appl Sci. (2022) 12:7934. doi: 10.3390/app12157934

[5] Ye P Liu Y Zhang J Peng K Pan X Shen Y. Falls prevention interventions for community-dwelling older people living in mainland China: a narrative systematic review. BMC Health Serv Res. (2020) 20:808. doi: 10.1186/s12913-020-05645-0, PMID: 32859186 PMC7456050

[6] Chantanachai T Sturnieks DL Lord SR Payne N Webster L Taylor ME. Risk factors for falls in older people with cognitive impairment living in the community: systematic review and meta-analysis. Ageing Res Rev. (2021) 71:101452. doi: 10.1016/j.arr.2021.101452, PMID: 34450352

[7] Deandrea S Lucenteforte E Bravi F Foschi R La Vecchia C Negri E. Risk factors for falls in community-dwelling older people: a systematic review and meta-analysis. Epidemiology. (2010) 21:658–68. doi: 10.1097/EDE.0b013e3181e89905 20585256

[8] Muir SW Gopaul K Montero Odasso MM. The role of cognitive impairment in fall risk among older adults: a systematic review and meta-analysis. Age Ageing. (2012) 41:299–308. doi: 10.1093/ageing/afs012, PMID: 22374645

[9] TsutsumimotoKDoiTMakizakoHHottaRNakakuboSMakinoK. Cognitive frailty is associated with fall-related fracture among older people. J Nutr Health Aging. (2018) 22:1216–20. doi: 10.1007/s12603-018-1131-4, PMID: 30498829

[10] Delbaere K Kochan NA Close JC Menant JC Sturnieks DL Brodaty H. Mild cognitive impairment as a predictor of falls in community-dwelling older people. Am J Geriatr Psychiatry. (2012) 20:845–53. doi: 10.1097/JGP.0b013e31824afbc4, PMID: 23011051

[11] Prosperini L Castelli L Sellitto G De Luca F De Giglio L Gurreri F. Investigating the phenomenon of "cognitive-motor interference" in multiple sclerosis by means of dual-task posturography. Gait Posture. (2015) 41:780–5. doi: 10.1016/j.gaitpost.2015.02.002, PMID: 25770078

[12] Giovannini S Iacovelli C Brau F Loreti C Fusco A Caliandro P. RObotic-assisted rehabilitation for balance and gait in stroke patients (ROAR-S): study protocol for a preliminary randomized controlled trial. Trials. (2022) 23:872. doi: 10.1186/s13063-022-06812-w, PMID: 36224575 PMC9558956

[13] Bai A Xu W Sun J Liu J Deng X Wu L. Associations of sarcopenia and its defining components with cognitive function in community-dwelling oldest old. BMC Geriatr. (2021) 21:292. doi: 10.1186/s12877-021-02190-1, PMID: 33957882 PMC8101237

[14] Xu W Chen T Shan Q Hu B Zhao M Deng X. Sarcopenia is associated with cognitive decline and falls but not hospitalization in community-dwelling oldest old in China: a cross-sectional study. Med Sci Monit. (2020) 26:e919894. doi: 10.12659/MSM.919894 31980594 PMC6998786

[15] Castelli L De Giglio L Haggiag S Traini A De Luca F Ruggieri S. Premorbid functional reserve modulates the effect of rehabilitation in multiple sclerosis. Neurol Sci. (2020) 41:1251–7. doi: 10.1007/s10072-019-04237-z, PMID: 31919697

[16] Giovannini S van der Roest HG Carfì A Finne-Soveri H Garms-Homolová V Declercq A. Polypharmacy in home Care in Europe: cross-sectional data from the IBenC study. Drugs Aging. (2018) 35:145–52. doi: 10.1007/s40266-018-0521-y, PMID: 29411310

[17] Huberty S Freystätter G Wieczorek M Dawson-Hughes B Kanis JA Rizzoli R. Association between multimorbidity and rate of falls: a 3-year 5-country prospective study in generally healthy and active community-dwelling adults aged ≥70 years. J Am Med Dir Assoc. (2023) S1525–8610:00971–9. doi: 10.1016/j.jamda.2022.12.011, PMID: 36657487

[18] Racey M Markle-Reid M Fitzpatrick-Lewis D Ali MU Gagne H Hunter S. Fall prevention in community-dwelling adults with mild to moderate cognitive impairment: a systematic review and meta-analysis. BMC Geriatr. (2021) 21:689. doi: 10.1186/s12877-021-02641-9, PMID: 34893027 PMC8665555

[19] Chen Y Zhang Y Guo Z Bao D Zhou J. Comparison between the effects of exergame intervention and traditional physical training on improving balance and fall prevention in healthy older adults: a systematic review and meta-analysis. J Neuroeng Rehabil. (2021) 18:164. doi: 10.1186/s12984-021-00917-0, PMID: 34819097 PMC8611920

[20] Xia L Zheng Y Lin Z Chen P Mei K Zhao J. Gap between risk factors and prevention strategies? A nationwide survey of fall prevention among medical and surgical patients. J Adv Nurs. (2022) 78:2472–81. doi: 10.1111/jan.15177 35293033 PMC9544575

[21] Tan JP Li N Gao J Wang LN Zhao YM Yu BC. Optimal cutoff scores for dementia and mild cognitive impairment of the Montreal cognitive assessment among elderly and oldest-old Chinese population. J Alzheimers Dis. (2015) 43:1403–12. doi: 10.3233/JAD-141278, PMID: 25147113

[22] BeauchetODubostVRevel DelhomCBerrutGBelminJ. How to manage recurrent falls in clinical practice: guidelines of the French Society of Geriatrics and Gerontology. J Nutr Health Aging. (2011) 15:79–84. doi: 10.1007/s12603-011-0016-6, PMID: 21267524

[23] Chu TK Chung JC. Psychometric evaluation of the Chinese version of the activities of daily living questionnaire (ADLQ-CV). Int Psychogeriatr. (2008) 20:1251–61. doi: 10.1017/S104161020800762X, PMID: 18664304

[24] Bottari CL Dassa C Rainville CM Dutil E. The IADL profile: development, content validity, intra- and interrater agreement. Can J Occup Ther. (2010) 77:90–100. doi: 10.2182/cjot.2010.77.2.5, PMID: 20464894

[25] Brañez-Condorena A Soriano-Moreno DR Navarro-Flores A Solis-Chimoy B Diaz-Barrera ME Taype-Rondan A. Accuracy of the geriatric depression scale (GDS)-4 and GDS-5 for the screening of depression among older adults: a systematic review and meta-analysis. PLoS One. (2021) 16:e0253899. doi: 10.1371/journal.pone.0253899, PMID: 34197527 PMC8248624

[26] Spitzer RL Kroenke K Williams JB Löwe B. A brief measure for assessing generalized anxiety disorder: the GAD-7. Arch Intern Med. (2006) 166:1092–7. doi: 10.1001/archinte.166.10.1092 16717171

[27] KaiserMJBauerJMRamschCUterWGuigozYCederholmT. Validation of the Mini nutritional assessment short-form (MNA-SF): a practical tool for identification of nutritional status. J Nutr Health Aging. (2009) 13:782–8. doi: 10.1007/s12603-009-0214-719812868

[28] Chen LK Liu LK Woo J Assantachai P Auyeung TW Bahyah KS. Sarcopenia in Asia: consensus report of the Asian working Group for Sarcopenia. J Am Med Dir Assoc. (2014) 15:95–101. doi: 10.1016/j.jamda.2013.11.025, PMID: 24461239

[29] Buysse DJ Reynolds CF 3rd Monk TH Berman SR Kupfer DJ. The Pittsburgh sleep quality index: a new instrument for psychiatric practice and research. Psychiatry Res. (1989) 28:193–213. doi: 10.1016/0165-1781(89)90047-4, PMID: 2748771

[30] Puhr R Heinze G Nold M Lusa L Geroldinger A. Firth's logistic regression with rare events: accurate effect estimates and predictions? Stat Med. (2017) 36:2302–17. doi: 10.1002/sim.7273, PMID: 28295456

[31] Imai K Keele L Tingley D. A general approach to causal mediation analysis. Psychol Methods. (2010) 15:309–34. doi: 10.1037/a0020761 20954780

[32] Rijnhart JJM Lamp SJ Valente MJ MacKinnon DP Twisk JWR Heymans MW. Mediation analysis methods used in observational research: a scoping review and recommendations. BMC Med Res Methodol. (2021) 21:226. doi: 10.1186/s12874-021-01426-3, PMID: 34689754 PMC8543973

[33] Kwan MM Close JC Wong AK Lord SR. Falls incidence, risk factors, and consequences in Chinese older people: a systematic review. J Am Geriatr Soc. (2011) 59:536–43. doi: 10.1111/j.1532-5415.2010.03286.x, PMID: 21361880

[34] Chu LW Chi I Chiu AY. Incidence and predictors of falls in the Chinese elderly. Ann Acad Med Singap. (2005) 34:60–72. PMID: 15726221

[35] Zhou R Li J Chen M. The association between cognitive impairment and subsequent falls among older adults: evidence from the China health and retirement longitudinal study. Front Public Health. (2022) 10:900315. doi: 10.3389/fpubh.2022.900315, PMID: 35784248 PMC9240660

[36] Allali G Launay CP Blumen HM Callisaya ML De Cock AM Kressig RW. Falls, cognitive impairment, and gait performance: results from the GOOD initiative. J Am Med Dir Assoc. (2017) 18:335–40. doi: 10.1016/j.jamda.2016.10.008, PMID: 27914848 PMC5366266

[37] Monachan D Vargese SS Johny V Mathew E. Risk of fall among older adults and its association with cognitive impairment in a semi-Urban Community. Indian J Community Med. (2020) 45:463–6. doi: 10.4103/ijcm.IJCM_491_19, PMID: 33623202 PMC7877416

[38] Ma Y Li X Pan Y Zhao R Wang X Jiang X. Cognitive frailty and falls in Chinese elderly people: a population-based longitudinal study. Eur J Neurol. (2021) 28:381–8. doi: 10.1111/ene.14572, PMID: 33030300

[39] Smith L Jacob L Kostev K Butler L Barnett Y Pfeifer B. Mild cognitive impairment is associated with fall-related injury among adults aged ≥65 years in low- and middle-income countries. Exp Gerontol. (2021) 146:111222. doi: 10.1016/j.exger.2020.111222, PMID: 33385480

[40] Liu S Wang F Zhang C Zhang Q Dang ZC Ng CH. Cognitive impairment and its associated factors in older adults living in high and low altitude areas: a comparative study. Front Psych. (2022) 13:871414. doi: 10.3389/fpsyt.2022.871414 PMC9259941 35815014

[41] McGrath R Vincent BM Hackney KJ Al Snih S Graham J Thomas L. Weakness and cognitive impairment are independently and jointly associated with functional decline in aging Americans. Aging Clin Exp Res. (2020) 32:1723–30. doi: 10.1007/s40520-019-01351-y, PMID: 31520335

[42] Cui M Zhang S Liu Y Gang X Wang G. Grip strength and the risk of cognitive decline and dementia: a systematic review and Meta-analysis of longitudinal cohort studies. Front Aging Neurosci. (2021) 13:625551. doi: 10.3389/fnagi.2021.625551, PMID: 33613270 PMC7890203

[43] Muhammad T Meher T. Association of late-life depression with cognitive impairment: evidence from a cross-sectional study among older adults in India. BMC Geriatr. (2021) 21:364. doi: 10.1186/s12877-021-02314-7 34130632 PMC8204463

[44] Muhammad T Sekher TV Srivastava S. Association of objective and subjective socioeconomic markers with cognitive impairment among older adults: cross-sectional evidence from a developing country. BMJ Open. (2022) 12:e052501. doi: 10.1136/bmjopen-2021-052501, PMID: 35981779 PMC9394209

[45] Liang F Fu J Turner-McGrievy G Wang Y Qiu N Ding K. Association of Body Mass Index and Plant-Based Diet with cognitive impairment among older Chinese adults: a prospective, Nationwide Cohort Study. Nutrients. (2022) 14:3132. doi: 10.3390/nu14153132 35956314 PMC9370436

[46] Nie XD Wang Q Zhang YH Xiong ZY Liao JL Hao L. Depression at baseline is an independent risk factor for cognitive decline in patients on peritoneal Dialysis: a multicenter prospective cohort study. Perit Dial Int. (2019) 39:465–71. doi: 10.3747/pdi.2018.00239, PMID: 31501292

[47] Cerveira MO Silva-da-Silva E Borelli WV Castilhos RM Chaves MLF. Association of Being Accompanied at medical consultation and having memory complaints with cognitive impairment in elderly Brazilian outpatients. Alzheimer Dis Assoc Disord. (2022) 36:295–9. doi: 10.1097/WAD.0000000000000521, PMID: 35867971

[48] Roh HW Lee DE Lee Y Son SJ Hong CH. Gender differences in the effect of depression and cognitive impairment on risk of falls among community-dwelling older adults. J Affect Disord. (2021) 282:504–10. doi: 10.1016/j.jad.2020.12.170, PMID: 33433379

[49] Lastrucci V Lorini C Caini S Bonaccorsi G. Health literacy as a mediator of the relationship between socioeconomic status and health: a cross-sectional study in a population-based sample in Florence. PLoS One. (2019) 14:e0227007. doi: 10.1371/journal.pone.0227007, PMID: 31869381 PMC6927637

[50] Chen LK Hwang AC Lee WJ Peng LN Lin MH Neil DL. Efficacy of multidomain interventions to improve physical frailty, depression and cognition: data from cluster-randomized controlled trials. J Cachexia Sarcopenia Muscle. (2020) 11:650–62. doi: 10.1002/jcsm.12534, PMID: 32134208 PMC7296266

[51] Jackson JW Vander Weele TJ Blacker D Schneeweiss S. Mediators of first-versus second-generation antipsychotic-related mortality in older adults. Epidemiology. (2015) 26:700–9. doi: 10.1097/EDE.0000000000000321, PMID: 26035686 PMC4720122

[52] Jang BJ Schuler MS Evans-Polce RJ Patrick ME. Marital status as a partial mediator of the associations between young adult substance use and subsequent substance use disorder: application of causal inference methods. J Stud Alcohol Drugs. (2018) 79:567–77. doi: 10.15288/jsad.2018.79.567, PMID: 30079872 PMC6090100

[53] Rijnhart JJM Valente MJ Smyth HL MacKinnon DP. Statistical mediation analysis for models with a binary mediator and a binary outcome: the differences between causal and traditional mediation analysis. Prev Sci. (2023) 24:408–18. doi: 10.1007/s11121-021-01308-6, PMID: 34782926 PMC9108123

