# Supplementary figures and images for: The direct and mediating effects of cognitive impairment on the occurrence of falls: a cohort study based on community-dwelling old adults
Source: Front Med (Lausanne). 2023 Jun 8;10:1190831. doi: 10.3389/fmed.2023.1190831 (PMC10285398; doi:10.3389/fmed.2023.1190831)

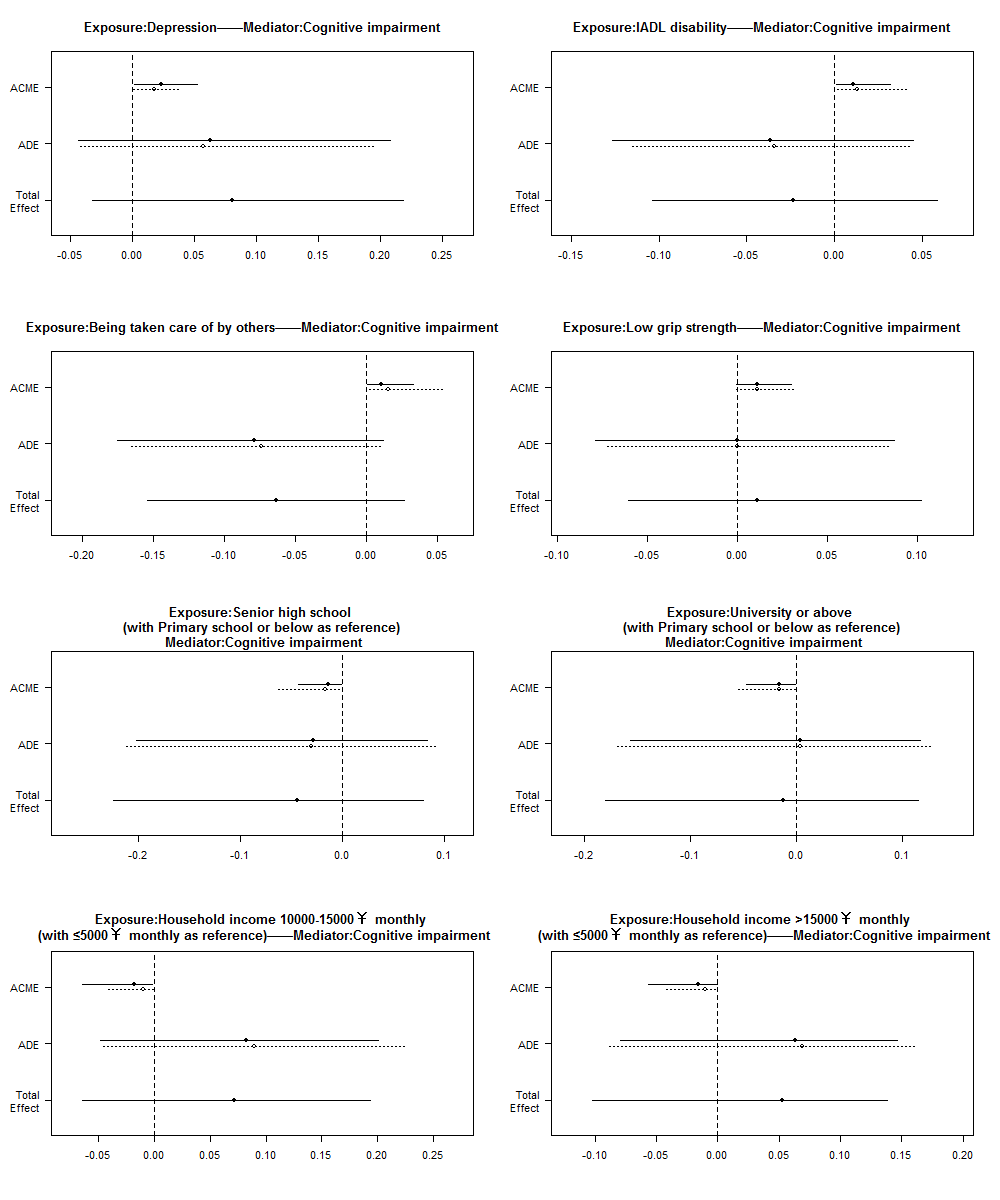

Supplement: SUPPLEMENTARY FIGURE S1 — The causal mediation analyses for fall, cognitive impairment and its associated factors. [file Image_1.TIFF]

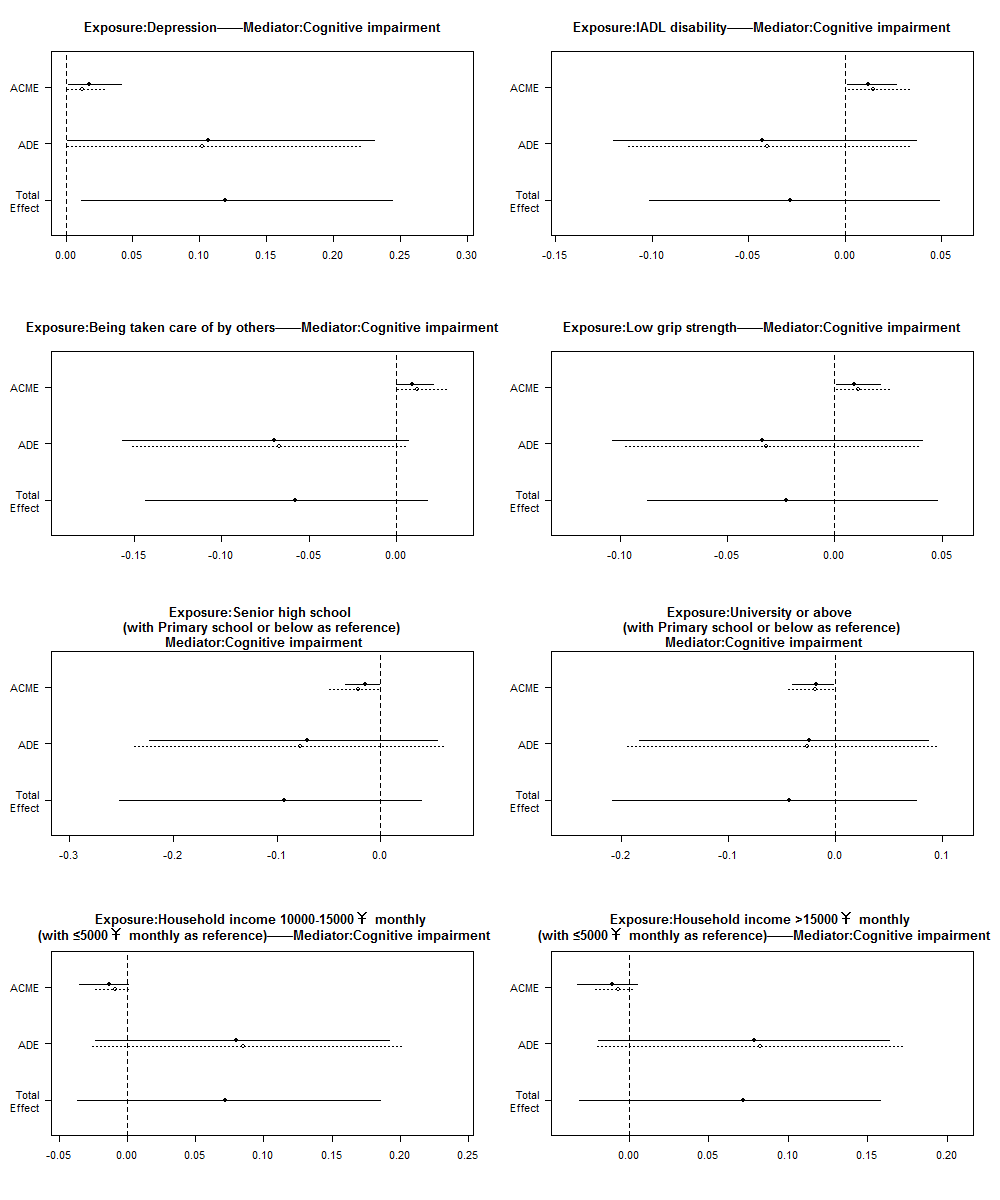

Supplement: SUPPLEMENTARY FIGURE S2 — The causal mediation analyses for fall, cognitive impairment and its associated factors after multiple imputation. [file Image_2.TIFF]
